# A panel of ten microsatellite loci for the Chagas disease vector *Rhodnius prolixus* (Hemiptera: Reduviidae)

**DOI:** 10.1016/j.meegid.2008.10.017

**Published:** 2009-03

**Authors:** S. Fitzpatrick, P.C. Watts, M.D. Feliciangeli, M.A. Miles, S.J. Kemp

**Affiliations:** a Pathogen and Molecular Biology Unit, Department of Infectious and Tropical Diseases, London School of Hygiene and Tropical Medicine, Keppel Street, London WC1E 7HT, UK; b School of Biological Sciences, The Biosciences Building, Crown Street, University of Liverpool, Liverpool, L69 7ZB, UK; c Facultad de Ciencias de la Salud, Universidad de Carabobo, Nucleo Aragua, and the Ministerio de Salud y Desarrollo Social, Instituto de Altos Estudios de Salud, Dr Arnoldo Gabaldon, Maracay, Venezuela

**Keywords:** *Rhodnius prolixus*, Chagas disease, Microsatellite panel, Gene flow, Triatominae, Venezuela, Colombia

## Abstract

*Rhodnius prolixus* is the main vector of Chagas disease in Venezuela, where it is found colonising rural housing consisting of unplastered adobe walls with palm and/or metal roofs. Vector control failure in Venezuela may be due to the invasion of houses by silvatic populations of *R. prolixus* found in palms. As part of a study to determine if domestic and silvatic populations of *R. prolixus* are isolated, thus clarifying the role of silvatic populations in maintaining house infestations, we constructed three partial genomic microsatellite libraries. A panel of ten dinucleotide polymorphic microsatellite markers was selected for genotyping. Allele numbers per locus ranged from three to twelve, with observed and expected heterozygosity ranging from 0.26 to 0.55 and 0.32 to 0.66. The microsatellite markers presented here will contribute to the control of Chagas disease in Venezuela and Colombia through the provision of population information that may allow the design of improved control strategies.

## 1 Introduction

Chagas disease is a chronic parasitic disease resulting from an infection of the protozoan *Trypanosoma cruzi*. Vectorial transmission by triatomine bugs (Reduviidae: Triatominae), is limited to the Americas. *Rhodnius prolixus* is the main vector of Chagas disease in Venezuela, where it is found infesting rural houses often formed from unplastered adobe walls with palm and/or metal roofs (Aché and Matos, 2001). Despite four decades of vector control using residual insecticide, domestic infestations of *R. prolixus* persist and transmission of *T. cruzi* may be increasing (Feliciangeli et al., 2003). This is in contrast to the Southern Cone initiative which has successfully eliminated *Triatoma infestans* from many endemic regions (Dias et al., 2002), with the notable exception of Bolivia. In addition to the domestic cycle research suggests *R. prolixus* may have a widespread silvatic distribution in palm trees in Venezuela (Gamboa, 1973). The invasion of houses from palm dwelling populations potentially explains these control failures. In the Bolivian Andes and the Gran Chaco (Bolivia and northern Argentina) silvatic populations of *T. infestans* also occur and may pose a risk to effective disease control in these regions (Noireau et al., 2005). For effective control it is necessary to study the degree of interaction between silvatic and domestic vector populations. If reinvasion commonly occurs then control programmes will have to be modified to deal with the silvatic threat. We developed a panel of ten polymorphic microsatellite markers for *R. prolixus* as part of a study to quantify the level of gene flow between silvatic and domestic *R. prolixus* populations in five Venezuelan States.

## 2 Materials and methods

Genomic DNA for library construction was isolated using a standard phenol chloroform protocol from the pooled legs of 11 field collected *R. prolixus* specimens. Three microsatellite libraries were created for the repeat motifs (1) CA/CAA, (2) GATA and (3) GAA/AAAG using a magnetic bead and biotinylated probe enrichment protocol based on Gardner et al. (1999), and provided elsewhere (Bloor et al., 2001). Briefly, a total of 9–20 μg of genomic DNA was digested with a blunt end restriction enzyme (*Sau*3AI, 24U). The restricted DNA fragments were then ligated to phosphorylated linkers (S61 5′-GGCCAGAGACCCCAAGCTTCG-3′ 25 pmol, annealed to S62 5′-PO_4_-GATCCGAAGCTTGGGGTCTCTGGCC-3′ 25 pmol, Refseth et al., 1997). Fragments were then size selected (400–1000 bp) and gel purified (QIAquick™ Qiagen). DNA fragments containing microsatellite motifs were captured as follows: streptavadin-coated magnetic beads (10 mg/ml M-280 Dynabeads, Dynal) were incubated with a 3′-biotin-labelled oligonucleotide probe (200 pmol CA/CAA, GATA or GAA/AAAG). Adaptor ligated DNA was incubated with the probe-coated beads at a specific hybridisation temperature. After several washes the enriched DNA was recovered and amplified by PCR. The PCR product was then purified (QIAquick Qiagen), ligated into pGEM^®^-T vector (Promega) and then transformed into JM109 *E. coli* competent cells (Promega). Recombinant clones were identified using black/white screening on S-gal (Sigma) agar/ampicillin plates. Recombinant colonies were screened by PCR as detailed in Bloor et al. (2001). Clones found to contain inserts were purified and sequenced.

The majority of clones screened (384, 66.7%) were isolated from library 1 (CA/CAA), 96 clones were screened for both libraries 2 and 3. Ninety-four positive clones were identified by PCR from the total of 576 screened. Ninety clones were sequenced and 84 were shown to contain microsatellite repeats (Beckman Ceq™ 2000, Beckman Coulter). Thirty-eight clones were not used for primer design: 9 had identical flanking regions (cluster analysis, BioEdit, Hall 1999), 1 was a false positive, 6 had flanking regions unsuitable for primer design and 22 clones required resequencing including 5 unconfirmed for repeat motifs. A total of 52 primer pairs were designed. The main design parameters were amplicon size (approximately 150–500 bp), *T*_M_ (≥40°) and lack of secondary structures (self dimers, primer dimers) (Primer3; Rozen and Skaletsky, 2000). Forty primers nestled dinucleotide CA repeats, while the remaining bordered varied repeat regions (see supplementary Table 1). Thirty-nine of the primer pairs amplified a single clean PCR band when tested.

Twenty-one primers flanking dinucleotide repeats were chosen for labelling with a 5′ fluorescent dye (6-FAM, PET, NED or VIC Applied Biosystems). Ten primer pairs were subsequently dropped due to inconsistent PCR-amplification or complex stutter patterns that became apparent after routine genotyping (see supplementary Table 1).

Microsatellite alleles were amplified by PCR in a total reaction volume of 10 μl containing approximately 10 ng extracted DNA, 5 pmol of each primer, 1 unit of *Taq* DNA polymerase (Bioline), 2 mM of each dNTP (Bioline), 1.5 mM MgCl_2_ (Bioline), 10 mM Tris–HCl pH 9.0, 50 mM KCL, 0.01% gelatin, 0.1% Triton X-100 (Bioline). PCR conditions were: (i) 1 min at 95 °C, (ii) 6 cycles of 30 s at 95 °C, 30 s at *T*_a_ °C and 45 s at 72 °C, (iii) 26 cycles of 30 s at 92 °C, 30 s at T_a_ °C and 55 s at 72 °C, and (iv) 72 °C for 30 min; *T*_a_ is the annealing temperature for each locus (Table 1). Cycle amplification was performed using a Primus 96 plus (MWG AG Biotech) or a PTC-100R (MJ research) thermal cycler. When a loci failed to amplify a maximum of 3 PCR attempts were made with adjusted *T*_M_ and MgCl_2_ levels. All PCR products were run with an internal size-standard (LIZ 500, Applied Biosystems) on an ABI 3730 48 capillary DNA analyser (Applied Biosystems). Allele fragment lengths were quantified using Genemapper software V3 (ABI).

We used Arlequin version 2.001 (Schneider et al., 2000) to calculate observed and expected heterozygosity and deviations from expected Hardy-Weinberg conditions (Guo and Thompson's (1992) Markov-chain random walk algorithm, 1000 iterations). The statistical significance of deviations from Hardy-Weinberg expectations was adjusted to maintain a type-I error rate of *α* = 0.05 using sequential Bonferroni correction (Rice, 1989). We also tested for linkage disequilibrium between all pairs of loci using the procedure implemented by Genepop (Raymond and Rousset, 1995).

## 3 Results

**Fig. 1:**
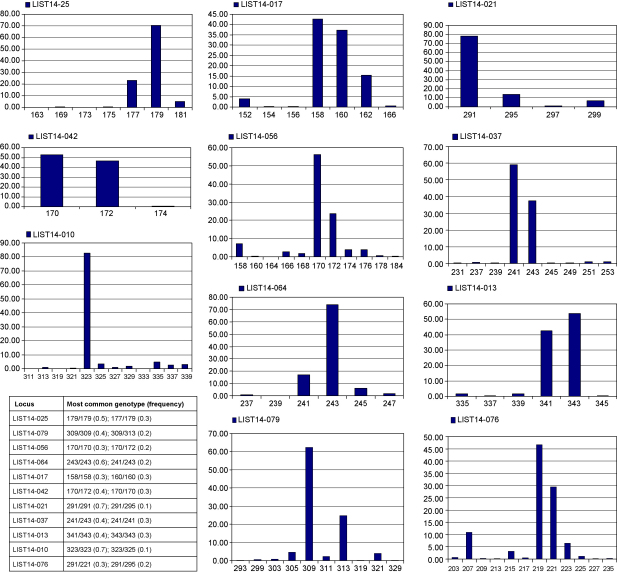
The distribution of alleles at each tested locus.

A total of 555 *R. prolixus* specimens collect from silvatic, domestic and peridomestic sites in five Venezuelan States were analysed using this panel of ten microsatellites. Results of the population analysis are reported elsewhere (Fitzpatrick et al., 2008). The number of alleles per locus ranged from 3 (LIST14-042) up to 12 (LIST14-010) (Table 1, Fig. 1). In this study 54 specimens failed to amplify at a single locus, when all other loci for these specimens generated product. Two specimens failed at two loci. All 10 loci experienced such non amplification, with greatest numbers occurring at LIST14-010, LIST14-037, LIST14-013 (11 and 10 specimens, respectively). Null alleles are only obvious in homozygotes where there is a failure to amplify product. Heterozygotes with a single null allelle could be mistakenly identified as homozygotes. Observed and expected heterozygosity varied between 0.26–0.55 and 0.32–0.66 respectively (Table 1). Seven loci showed significant deviations from expected Hardy-Weinberg conditions after sequential Bonferroni due to heterozygosity deficiency. However this might be expected as specimens were pooled from 33 populations from 5 States in Venezuela. The number of polymorphic loci in populations ranged from 6 to 10, with 85% of all populations polymorphic at all loci. Fisher's exact test across each pair of loci in the pooled populations detected significant linkage at two locus pairs, LIST14-056 with LIST14-013 and LIST14-056 with LIST14-076, both remained significant after Bonferroni correction. Significant linkage disequilibrium was detected in 96 locus pairs in 30 populations, however only 18 pairs remained significant after sequential Bonferroni correction: LIST14-017 with LIST14-042 (1 population), LIST14-010 with LIST14-013 (1 population), LIST14-010 and LIST14-025 (1 population) and LIST14-056 with LIST14-076 (15 populations). Due to the consistent linkage pattern exhibited by LIST14-056 with LIST14-076, locus LIST14-076 was excluded from further population analysis (Fitzpatrick et al., 2008). Cluster analysis of all 10 loci did not detect shared flanking regions (BioEdit, Hall 1999).

## 4 Discussion

Using this panel of microsatellites we detected gene flow among silvatic, domestic and peridomestic populations in Venezuela (Fitzpatrick et al., 2008). We demonstrated that silvatic invasion by *R. prolixus* represents a definite threat to Chagas disease control, as suspected but debated since populations of *R. prolixus* were reported in palm trees. The current control programme in Venezuela is unlikely to achieve the level of success seen in the Southern Cone and a more suitable control strategy may include frequent spraying of houses, combined with community vigilance for reinfestations as an integral part of the control programme.

An additional ten microsatellite loci have recently been published for *R. prolixus* and while tested on only five colony specimens to date, these markers should also prove highly informative for the analysis of natural populations (Harry et al., 2008), particularly in regions of Colombia, where silvatic and domestic *Rhodnius* populations also occur and reinvasion may be maintaining domestic colonies of *R. prolixus* (Guhl et al., 2005). This would allow prioritisation of control interventions and tailoring of control strategies to regional circumstances.

## Figures and Tables

**Table 1:** Characterisation of ten polymorphic microsatellite loci amplified from field collected *Rhodnius prolixus*.

Locus	Primer sequence (5′ → 3′)	Repeat array	Dye	*T*_a_	Allele size range (bp)	*N*	*N*_a_	*A*_F_	*A*_N_	*N*_G_	*H*_o_ (*H*_e_)	HWE
GenBank accession												
LIST14-056	F: TTTCCATTTGGCTCGTTTTGC	[CA]_16_N_14_[CT]_6_	PET	57	158–184	555	11	0.56	4	30	0.55	<0.0001
EU622033	R: GATAGTGCGATACATTTTGC										(0.62)	
LIST14-017	F: ATTGAAGGTTACTACTTGCTGC	[TG]_12_	FAM	57	152–166	555	7	0.43	5	15	0.31	<0.0001
EU622028	R: ACGCTGCTTCATTTTTTAGTGG										(0.66)	
LIST14-042	F: TACTTCCGACTGACAACCG	[GT]_9_	FAM	50	170–174	555	3	0.53	3	5	0.40	0.0003
EU622031	R: GGTTTTAGTTCACCAATAGC										(0.50)	
LIST14-010	F: AATGATGACTGTATTGATGGGC	[CA]_9_	FAM	52	311–339	555	12	0.83	11	31	0.26*	<0.0001
EU622026	R: TTCGACCAACAACAACTTCCC										(0.32)	
LIST14-064	F: AGAAAATGAGCAAAACGGCC	[GT]_10_	FAM	57	237–247	555	6	0.74	5	13	0.27	<0.0001
EU622035	R: ACAGGCAAACAACTATGACG										(0.42)	
LIST14-013	F: CATACTACACGCACACAAGACC	[AC]_10_	PET	55	335–345	555	6	0.54	10	12	0.50	0.062
EU622027	R: ATACTCGCATCAAGCCATTTGG										(0.53)	
LIST14-021	F: AACCTCTGAACACATCAAATGG	[TG]_10_	NED	55	291–299	555	4	0.78	1	10	0.27	<0.0001
EU622029	R: AGCTACCTCTTGCCTCTACG										(0.37)	
LIST14-037	F: GGCGACACCCCATAGAAACC	[GT]_8_	PET	55	231–253	555	9	0.59	10	15	0.51	0.095
EU622032	R: ATTAAAGAACGGAAACCCCACC										(0.51)	
LIST14-076	F: AGATAGTGCGATACATTTTGCG	[AG]_6_N_14_[TG]_17_	FAM	52	203–235	305	12	–	3	25	–	
EU622025	R: GTTAGAGTTGTCCTCAAGAAGC											
LIST14-025	F: CCGCTCTATCAACTACTCC	[TC]_9_[AC]_7_N_13_[AC]_7_	NED	50	163–181	555	7	0.71	6	10	0.43	0.010
EU622030	R: GATCCCTTATGTTTCTCAGC										(0.44)	
LIST14-079	F: TAGAGTTTTTGCTCCTGTTAGC	[CA]_9_N_2_[CA]_10_	FAM	52	293–329	305	10	0.62	1	9	0.41	<0.0001
EU622034	R: TCCTATCTTTCGGTAAGTCCG										(0.55)	

*T*_a_, annealing temperature of the primer pair (°C); *N*, number of specimens amplified for this study; *N*_a_, number of alleles; *A*_F_, frequency of the most common allele; *A*_N_, number of null alleles detected per locus, A locus was considered null after a maximum of three PCR attempts with adjusted temperature and MgCl_2_ levels; *N*_G_, number of genotypes detected per locus; *H*_o_, observed heterozygosity; *H*_e_, expected heterozygosity. *P*-value-exact probability for expected Hardy-Weinberg equilibrium conditions for each locus/population combination (Arlequin v2.1).
