# Supplementary material for: A panel of ten microsatellite loci for the Chagas disease vector Rhodnius prolixus (Hemiptera: Reduviidae)
Source: Infect Genet Evol. 2009 Mar;9(2):206–9. doi: 10.1016/j.meegid.2008.10.017 (PMC2651431; doi:10.1016/j.meegid.2008.10.017)
Supplement: Supplementary file 1 [file mmc1.doc]

**Supplementary** **Table 1**: All 52 primer pairs designed from three enriched microsatellite *R. prolixus* libraries

| Primer sequence | Sequence (5'-3') | Cloned allele  size (bp) | Repeat motif | Annealing temp (C°) |
| --- | --- | --- | --- | --- |
| LIST14-001F | CCC AAT ACA ACA CCC AAT ACC | ** | [GT] | 52 |
| LIST14-001R | GTG ACG GTG CCA TGT TAG G |  |  |  |
| LIST14-002F | GCT GAA AAT GAG CAA AAA CGG | 211 | [CTT] N16 [GTT]6 | 53 |
| LIST14-002R | ATC GAG ACC CCC AAA AGG C |  |  |  |
| LIST14-003F | ACT TCT CTT GCC TTT GAC TCC | 189 | [CTT]7[TCT]18 | 54 |
| LIST14-003R | CGG AAG GAA GTC TAA AAC TCG |  |  |  |
| LIST14-004F | ATA CCT ACC CAG TAA AAA GC | 211 | [GT]18 | 52 |
| LIST14-004R | GAG AAG TTG AAA AGT TGA CC |  |  |  |
| LIST14-005F | TCG GTT CAC GCG TTT AGG CG | 233 | [CAA]5[GAA]12 | Failed at 50 52 53 55 |
| LIST14-005R | GAT CTC AGG AGG TAG TTC AGG |  |  |  |
| LIST14-007F | CTTTTCCGTCTTGCAGGAAGG | 185 | [CT]16 [CA]14 | Inconsistent amplification * |
| LIST14-007R | CCGGTTGACCCGTTAGTTGG |  |  |  |
| LIST14-009F | ATGGTAAAAGTCGCAAAGCCGG | 213 | [GT]12[AT]2 | 55 |
| LIST14-009R | TTTCTGCTAAAGCTGTGCCCGG |  |  |  |
| LIST14-010F | AATGATGACTGTATTGATGGGC | 322 | [CA]9 | 55 |
| LIST14-010R | TTCGACCAACAACAACTTCCC |  |  |  |
| LIST14-013F | CATACTACACGCACACAAGACC | 341 | [AC]^10^ | 55 |
| LIST14-013R | ATACTCGCATCAAGCCATTTGG |  |  |  |
| LIST14-014F | TTCTGTTTCTCTGATTCCAGG | 301 | [CA]18 | Complex stuttering * |
| LIST14-014R | ACGTGTTGTGGTCTCTCG |  |  |  |
| LIST14-016F | ATAATACTAAAGGTGCCGATGG | 206 | [AC]24 | Failed at 50 52 53 55 |
| LIST14-016R | TGTATTGTCTCAGTTGAACACC |  |  |  |
| LIST14-017F | ATTGAAGGTTACTACTTGCTGC | 161 | [TG]12 | 55 |
| LIST14-017R | ACGCTGCTTCATTTTTTAGTGG |  |  |  |
| LIST14-019F | CTCTGTTAGTAGATTGTGGAGG | 149 | [GT]11 | Multiple banding in PCR * |
| LIST14-019R | CGCAACTGCTTTGGGTTTAGC |  |  |  |
| LIST14-021F | AACCTCTGAACACATCAAATGG | 297 | [TG]10 | 55 |
| LIST14-021R | AGCTACCTCTTGCCTCTACG |  |  |  |
| LIST14-025F | CCGCTCTATCAACTACTCC | 180 | [TC]^9^[AC]7N8[AC]7 | 50 |
| LIST14-025R | GATCCCTTATGTTTCTCAGC |  |  |  |
| LIST14-028F | AAATAGAGCAGCGTTGGACG | 293 | [TG]19 | 55 |
| LIST14-028R | CTTGCAGACAGGGAATCACC |  |  |  |
| LIST14-029F | ATCAAGCTGAACGCCTTAGG | 300 | [TG]25 | 55 |
| LIST14-029R | TCAGCATAGTTAGGATGGAACC |  |  |  |
| LIST14-031F | AGAGAGCGTAGAAGTGGC | 269 | [AC]17 N2[AC]8 N2 [AC]6 | 50 |
| LIST14-031R | TTCGGGTCCGTAGTTTGG |  |  |  |
| LIST14-032F | GTTGTCCAGCACTTTGTTGG | 248 | [GT]22 | Complex stuttering * |
| LIST14-032R | TTTTTAGTAGGCTTGTAGGC |  |  |  |
| LIST14-035F | TTACAGATAAAACAGTAGCCGC | 218 | [TG]12 | 55 |
| LIST14-035R | GGTGTCCCATCCTAACATCG |  |  |  |
| LIST14-037F | GGCGACACCCCATAGAAACC | 239 | [GT]8 | 55 |
| LIST14-037R | ATTAAAGAACGGAAACCCCACC |  |  |  |
| LIST14-039F | ATTGAAGGTTACTACTTGCTGC | 161 | [TG]12 | Multiple banding in PCR * |
| LIST14-039R | ACGCTGCTTCATTTTTTAGTGG |  |  |  |
| LIST14-041F | CCAATACAACACATACACTCG | 160 | [CA]17 | Complex stuttering * |
| LIST14-041R | ATCTGACACGACGTGATTCC |  |  |  |
| LIST14-042F | TACTTCCGACTGACAACCG | 170 | [GT]9 | 50 |
| LIST14-042R | GGTTTTAGTTCACCAATAGC |  |  |  |
| LIST14-044F | CTTATGTTTGCTCAGAGGC | 242 | [TG]15 N2 [TTG]8 | Failed at 50 52 53 55 c |
| LIST14-044R | AGAAGGCCAGCCATTTCC |  |  |  |
| LIST14-047F | CACTCGTATCCGAATATAGC | 225 | [CA]19 | 55 |
| LIST14-047R | GGATGAAGAATGTTGCGGTGG |  |  |  |
| LIST14-050F | CGGACACATTTTCGGTTGG | 201 | [CA]8 | Multiple banding in PCR * |
| LIST14-050R | ATACAAGTTTTGAAGCCACC |  |  |  |
| LIST14-052F | GAAAACTGAAGATGAAGAAGGC | 288 | [AC]10 | Failed at 50 52 53 55 c |
| LIST14-052R | GAGGACAATAGGCCTGTGTGG |  |  |  |
| LIST14-053F | GACCAAGCAGATAGATAGC | 127 | [GT]15 | Failed at 50 52 53 55 c |
| LIST14-053R | AGAAGGCCAGCCATTTCC |  |  |  |
| LIST14-055F | CTTATAGAATGGAGACGTCC | 157 | [GT]23 | Multiple banding in PCR * |
| LIST14-055R | CAGAGGTAGTTGATGTGTGC |  |  |  |
| LIST14-056F | TTTCCATTTGGCTCGTTTTGC | 167 | [CA]16 N14 [CT]6 | 50 |
| LIST14-056R | GATAGTGCGATACATTTTGC |  |  |  |
| LIST14-061F | ATTTAGTGGACCAACCTCTAGC | 91 | [TG]13 | Multiple banding in PCR * |
| LIST14-061R | CTCCTACAACCATTCCGCCC |  |  |  |
| LIST14-064F | AGAAAATGAGCAAAACGGCC | 242 | [GT]10 | 50 |
| LIST14-064R | ACAGGCAAACAACTATGACG |  |  |  |
| LIST14-067F | CTGTGGAAAGCGACTCCCTGG | 198 | [AC]12 | Multiple banding in PCR * |
| LIST14-067R | GCTTTGGGCACCTGGCAGATGG |  |  |  |
| LIST14-069F | TCGCTTTATTGTTAGGTAGGGG | 342 | [GAA]9 | Shared flanking region |
| LIST14-069R | TGCCGAAAATGAGCAAAAACGG |  |  | with LIST14-072 |
| LIST14-072F | ACATAAAGGGGGCTAACTCC | 258 | [GAA]16 | multiple bands 50-55 |
| LIST14-072R | CGAAAATGAGCAAAAACGGC |  |  |  |
| LIST14-073F | ACCAGCGTCCTTTTAAATGACG | 186 | [GT]13 | failed at 53 |
| LIST14-073R | TGCAGAATCCTCACACAATACC |  |  |  |
| LIST14-074F | TTCTCATTGGGCAAAATACC | 299 | [CT]6 N22 [TG]14 | 53 |
| LIST14-074R | GCAAAACATTCCTGATAACC |  |  |  |
| LIST14-075F | GCCCCTAAAAAATCTTGAATGC | 131 | [TG]16 | 53 |
| LIST14-075R | AAATAACCGCTCGGCTACCG |  |  |  |
| LIST14-076F | AGATAGTGCGATACATTTTGCG | 218 | [TG]17 | 55 |
| LIST14-076R | GTTAGAGTTGTCCTCAAGAAGC |  |  |  |
| LIST14-077F | GCACACTAAATACCACTGAGG | 284 | [GT]9 | 55 |
| LIST14-077R | AACCCTGTCACCACTACACACG |  |  |  |
| LIST14-078F | GCAGTTCTATGGAATCTCC | 292 | [GT]18 | 53 |
| LIST14-078R | TAAGGCGTGACATTAGTGC |  |  |  |
| LIST14-079F | TAGAGTTTTTGCTCCTGTTAGC | 314 | [CA]7 N2 [CA]10 | 53 |
| LIST14-079R | TCCTATCTTTCGGTAAGTCCG |  |  |  |
| LIST14-080F | CGCTTTAATGTAACGTAGGGG | 366 | [GAA]15 | multiple bands 50-60 |
| LIST14-080R | CCGAAAATGAGCAAAAACGGC |  |  |  |
| LIST14-081F | GCAAAACAGCAACAAACACACC | 95 | [CA]12 | failed at 53 |
| LIST14-081R | ATACAAAACACGGGTTATCTCG |  |  |  |
| LIST14-082F | CGTCACCCATACTTCAGAGG | 242 | [AC]10 | failed at 55, 57 |
| LIST14-082R | ATTGGCCAGAGACCCCAAGC |  |  |  |
| LIST14-083F | ATAATACTAAAGGTGCCGATGG | 269 | [CA]17 | 55 |
| LIST14-083R | GATCTGACACGACGTGATTCC |  |  |  |
| LIST14-084F | AGTTTTGAAGCCACCTGTGTGC | 305 | [GT]11 | failed at 50-60 |
| LIST14-084R | ATTGGTAGTTGGACGATAAGCC |  |  |  |
| LIST14-085F | AAAGTCTGACACTACCGCAGG | 165 | [AC]31 | 52 |
| LIST14-085R | CCTGATTGATACATACAGCACC |  |  |  |
| LIST14-086F | TACAGGAGGCGCAGTTAGTGG | 320 | [CA]17 | 57 |
| LIST14-086R | ATGCACCGCTTCTATTCAACCG |  |  |  |
| LIST14-087F | CTTGTTCCTATTGCTAAACTGG | 254 | [CA]17 | 55 |
| LIST14-087R | CTCTGCCAATCAAATTCTTAGC |  |  |  |
| LIST14-088F | GCAGTTCTATGGAATCTCC | 145 | [GT]18 | failed at 50-60 |
| LIST14-088R | GAGTCCCAGTTATTTACAGC |  |  |  |

** File misplace exact number repeats unknown; * Primer problems detected following fluorescent dye labeling and primer subsequently dropped.
